# Commercial implementation of genomic selection in Tasmanian Atlantic salmon: Scheme evolution and validation

**DOI:** 10.1111/eva.13304

**Published:** 2021-10-11

**Authors:** Klara L. Verbyla, Peter D. Kube, Bradley S. Evans

**Affiliations:** ^1^ CSIRO, Black Mountain Canberra ACT Australia; ^2^ CSIRO Hobart Tas. Australia; ^3^ Tassal Operations Hobart Tas. Australia; ^4^ Present address: Center for Aquaculture Technology San Diego CA USA

**Keywords:** Atlantic salmon, breeding, commercial implementation, genomic prediction

## Abstract

Genomic information was included for the first time in the prediction of breeding values for Atlantic salmon within the Australian Salmon Enterprises of Tasmania Pty Ltd selective breeding program in 2016. The process to realize genomic selection in the breeding program begun in 2014 with the scheme finalized and fully implemented for the first time in 2018. The high potential of within family selection to accelerate genetic gain, something not possible using the traditional pedigree‐based approach, provided the impetus for implementation. Efficient and effective genotyping platforms are essential for genomic selection. Genotype data from high density arrays revealed extensive persistence of linkage disequilibrium in the Tasmania Atlantic salmon population, resulting in high accuracies of both imputation and genomic breeding values when using imputed data. Consequently, a low‐density novel genotype‐by‐sequence assay was designed and incorporated into the scheme. Through the use of a static high‐ and dynamic low‐density genotyping platforms, an optimized genotyping scheme was devised and implemented such that all individuals in every year class are genotyped efficiently while maximizing the genetic gains and minimizing costs. The increase in the rates of genetic gain attributed to the implementation of genomic selection is significant across both the breeding programs primary and secondary traits. Substantial improvement in the ability to select parents prior to progeny testing is observed across multiple years. The resultant economic impacts for the industry are considerable based on the increases in genetic gain for traits achieved within the breeding program and the use of genomic selection for commercial production.

## 1 INTRODUCTION

Genomic selection has been widely deployed to improve the rate of genetic gain across a number of species (Cleveland & Hickey, 2013; Crossa et al., 2017; Hayes et al., 2009; Lin et al., 2014). Genomic selection is a genetic improvement strategy that utilizes genome‐wide molecular markers to capture all sources of genetic variation allowing for selection of individuals based on their total genetic value. This approach relies on having a sufficient number of markers located across the genome that are in linkage disequilibrium with all quantitative trait loci or genes linked to the traits of interest. Genomic selection is especially useful for complex and quantitative traits controlled by many genes with small or moderate effects, hard to measure traits and complex breeding strategies where phenotypes are only measured on subsets of the population.

Recently, there have been advances in the development and application of genomic selection for genetic improvement in aquaculture species (Boudry et al., 2021; Houston et al., 2020; Lhorente et al., 2019; Norris, 2017; Regan et al., 2021; Symonds et al., 2019; Zenger et al., 2019), including Atlantic salmon (*Salmo salar*) (Lhorente et al., 2019; Regan et al., 2021). The use of genomic selection in aquaculture breeding programs has the potential to drive high rates of genetic gain, while also controlling inbreeding and diversity, due to the high fecundity of species and potentially low effective population sizes within breeding programs. Genomic selection's reliance on genome‐wide markers has meant there has been focus on the development of genomic resources across a range of aquaculture species (Abdelrahaman et al., 2017; Houston et al., 2014; Yanez et al., 2016).

The Australian salmon industry began in 1986 with a first harvest of 50 tonnes and has grown to an annual production of approximately 70,000 tonnes in 2020. All Australian Atlantic salmon farming occurs in Tasmania, where waters are among the warmest in the world for Atlantic salmon culture. The warm temperatures allow Tasmanian Atlantic salmon to grow to a harvestable size significantly faster than in other farmed areas. However, faster growth can have problematic consequences. These include early sexual maturation, which has adverse effects on carcass quality and product availability, and temperature‐related susceptibility to disease, notably amoebic gill disease (AGD), that can result in significant stock losses. Consequently, harvest weight, AGD disease resistance, and maturation incidence are all recognized as important traits for the Tasmanian salmon industry.

Since inception, the industry has been quick to recognize that neither the environmental conditions nor the stock that is farmed are identical to those in other parts of the world. Consequently, systems and processes continue to be optimized for a Tasmanian context. Early research examined topics ranging from smoltification, lighting, maturation, ploidy levels, sex ratios, and smolt input. The development of the Tasmanian breeding program mirrors this progression, with the development and incorporation of innovations in response to both global and uniquely Tasmanian issues. The implementation of the breeding program and the desire to push gains using genomic selection represent a progression of that innovation.

This paper details the transition of the Tasmanian Atlantic salmon selective breeding program from conventional family‐based breeding to genomic selection. The aim is to provide a comprehensive overview of the steps taken to achieve commercial implementation with sufficient detail to enable the information to be presented in a single reference. This is the first time all aspects of such a process have been specifically outlined and addressed for an aquaculture species in a commercial breeding program. First, the breeding program is overviewed, detailing the population structure, breeding objectives, and the program logistics. Second, the process of developing genotyping tools and a breeding strategy is described, culminating in the full commercial implementation of genomic selection. Third, the validation and rates of genetic gain are reported. Finally, the challenges, results, and learnings from the implementation and use of genomic selection in the Tasmanian salmon industry are discussed.

## 2 SELECTIVE BREEDING PROGRAM OVERVIEW

The Tasmanian Atlantic salmon breeding program commenced in 2004 and, since then, has been a family‐based program producing a cohort of pedigreed families annually. It is now an advanced generation breeding program with up to 6 generations of known pedigree in some lineages. It is owned and operated by Salmon Enterprises of Tasmania Pty Ltd (SALTAS). Founders were deliberately sourced for diversity from the Tasmanian Atlantic salmon landrace, which originates from wild stocks imported from River Philip, Nova Scotia, Canada. Stock was imported as ova to Gaden, New South Wales, in 1963 to 1965 and then moved to Tasmania in 1984–1986 when salmon farming commenced (Ward et al., 1994).

The breeding objective has identified primary traits, secondary traits and monitoring traits (Table 1), and the breeding goal has been to maximize gains equally in each of the primary traits without causing adverse change in secondary traits. One primary trait is harvest weight, measured as a gutted weight at approximately 28 months from spawning (or after 14 to 15 months marine grow‐out), and this is a typical trait for all salmon breeding programs. The other primary trait is resistance to amoebic gill disease (AGD), and the strong emphasis and selection for this trait are probably unique to this breeding program. Progress toward the breeding goal for AGD is expressed as an increase in the treatment interval (freshwater baths) during the marine grow‐out. Earlier work on AGD resistance recognized two distinct traits: one, termed innate AGD resistance, is measured at first infection on naïve fish and has a low heritability; and the other, termed acquired AGD resistance, is measured at all subsequent infections and has a moderate heritability (Kube et al., 2012). Acquired AGD resistance has much higher economic value than innate AGD resistance, and acquired AGD resistance is the primary trait in the breeding objective. Table 1 shows the accumulated and average annual gains for each trait, from inception to the 2015 year class, which is the period of traditional family‐based breeding prior to the commencement of genomic selection.

**TABLE 1:** Breeding objectives, heritabilities (h2), and genetic gains for the Tasmanian Atlantic salmon breeding program

Trait type	Trait	h2 (se)	Selection pressure	Cumulative gains to 2015 YC	Mean annual gains	Model
Primary	Acquired AGD resistance	0.36 (0.02)	38%	29%	3.7%	AGD
Primary	Harvest weight	0.44 (0.02)	25%	29%	3.6%	Weight
Secondary	Innate AGD resistance	0.16 (0.01)	13%	0%	0%	AGD
Secondary	Marine maturation	0.20 (0.02)	13%	2%	0.2%	Maturation
Secondary	Flesh color	0.65 (0.04)	13%	7%	0.9%	Quality
Monitor	Flesh fat content	0.28 (0.03)	0%	−5%	−0.6%	Quality

Note

Genetic gains are expressed as improvement from founder stocks (Tasmanian landrace) and are calculated routinely via the EBV/GEBV mixed models. Gains are expressed such that a positive value represents a favorable change and a negative value an unfavorable change. Heritabilities (h2) were calculated as part of the routine EBV calculations and used the models described in Section 3.5.

The broodstock population is maintained in a biosecure freshwater hatchery at Wayatinah in central Tasmania and, annually, 1.25‐year‐old smolt are transferred to two marine sites in southeastern Tasmania for grow‐out and assessment. Marine tests occur in purpose‐built pens that contain approximately 2500 individuals at stocking. Repeated measures of AGD resistance are taken (see Kube et al., 2012 for methods) together with marine maturation at age 2 years and finally harvest weight and carcass quality at 2.5 years. A further 3000 individuals are kept in freshwater as broodstock candidates, with total weight and maturation measurements being recorded for these animals. Marine animals do not return to the hatchery due to the biosecurity requirements and, therefore, broodstock have no marine phenotypes. Selections for harvest weight and marine maturation benefit through moderately high genetic correlations between freshwater and marine measurements (*r_g_* = 0.63 ± 0.03 and 0.76 ± 0.04, respectively); however, there are no significant genetic correlations between AGD resistance and freshwater traits. Consequently, AGD selections have been made via pedigree relationships alone.

Approximately 200 families have been produced every year using a partial factorial mating design where each male and female were crossed twice. The generation interval is notionally three years but the age at which broodstock were used has been variable. Males are used between the ages of 2–6 years and females from 3 to 6 years. The population is managed and analyzed as a single population by using genetic links between year classes. Genetic links occur using male and female individuals as repeat spawners in multiple year classes (about 15% of broodstock in any year class) together with the higher order genetic relationships (e.g., siblings or cousins used in subsequent years as broodstock) that naturally occur through using overlapping generations. Repeat spawners are used as both live animals and as cryopreserved sperm. All broodstock, whether they be designated genetic links or individuals from older year classes, are selected on genetic merit. Genetic evaluations are done using multivariate individual animal models (Section 3.5) in ASReml (Gilmour et al., 2015) to calculate estimated breeding values (EBV). These models use the BLUP methodology and, consequently, routinely calculate the variance components, which were used for the heritability estimates (Table 1), and they provide a straightforward means to calculate the genetic gains (Isik et al., 2017), which are used throughout this paper.

This breeding program has used genotyping to determine pedigree relationships since inception. Families are fertilized separately and pooled at the eyed‐egg stage when 250 eyed‐eggs are removed from each family to form a pooled group of approximately 50,000 individuals, which are then communally reared. At 12 months, approximately 8000 presmolt are randomly selected from this group for use as the freshwater broodstock and the marine test individuals. All 8000 individuals are, tagged using passive integrated transponder (PIT) tags, biopsied by removing a piece of caudal fin, and then genotyped for pedigree determination. Broodstock are re‐biopsied at spawning and the genotypes from these parents, plus the genotypes from the progeny, are used for pedigree determination.

**FIGURE 1:**
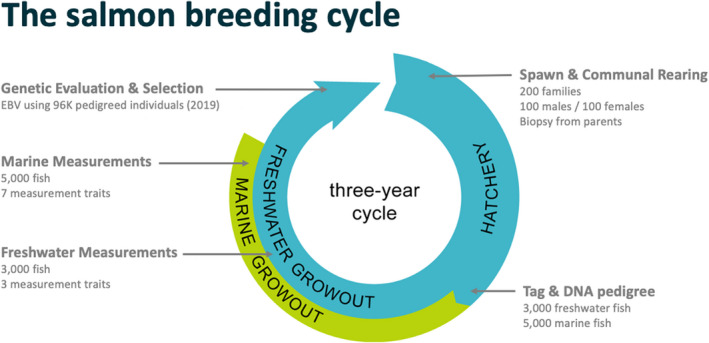
The traditional salmon breeding cycle

The typical cycle of operations is illustrated in Figure 1. The figure shows the cycle for a single year class but, in practice, three year classes are in operation at any point in time. Therefore, activities shown on Figure 1 form an annual work plan that involves family spawning, tagging, marine and freshwater measurements, genetic evaluation, and selection, and with some of those annual activities occurring on different year classes. All operations are time‐dependent, timelines are often short, and timing is inflexible since it is dictated by the biological cycle of the animal. These considerations were always important with a conventional selection program and become even more critical when considering the incorporation of new innovations and technologies, such as genomic selection.

## 3 GENOMIC SELECTION STRATEGY EVOLUTION

**FIGURE 2:**
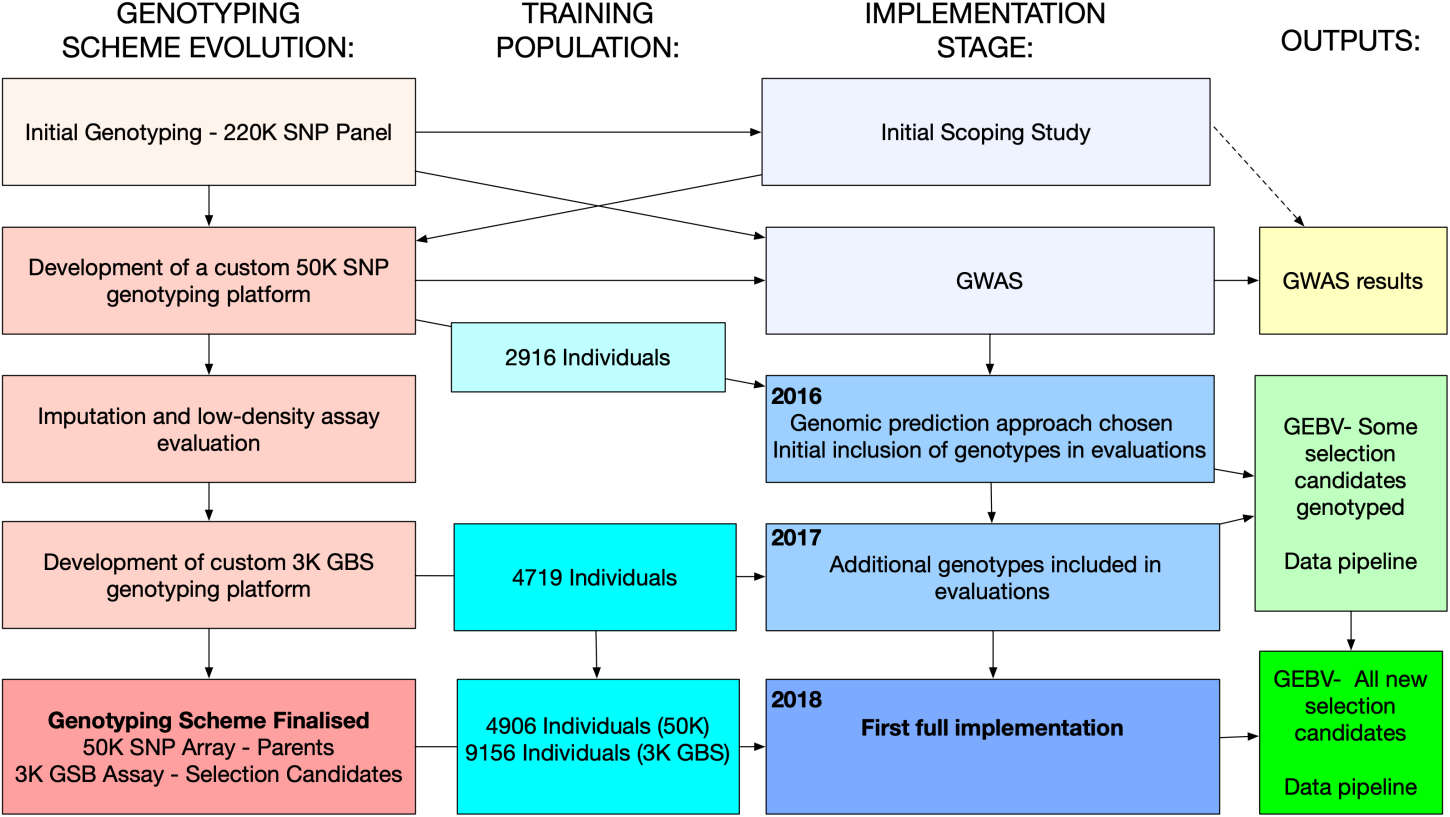
Genomic selection scheme stages of development

Genomic selection was first proposed as an opportunity within the SALTAS selective breeding program in 2010 where the business case and value proposition for its use to improve the rate of gains for AGD resistance was examined. Acceleration of studies scoping the potential of genomic selection occurred in 2014 with the reception of the first set of high‐dimensional genomics data. From that point on, the evolution of the genomic selection strategy and genotyping scheme involved multiple stages and these are shown in Figure 2. This involved building a robust training population (marine test individuals) and undertaking a number of key activities, described in the following sections, before full commercial implementation was achieved in 2018. Key aspects of the evolution are briefly described in the following sections with a focus on the development of the cost‐effective and customized genotyping scheme. The validation results for the implementation of genomic selection are presented in Section 4.

### 3.1 220K array analysis

In order to assess the feasibility of genomic selection for the Tasmanian breeding population and the genotyping tools requirements for the successful implementation, high‐density genotype data were sourced using a custom 220K SNP Affymetrix array developed by AquaGen and CIGENE (Barson et al., 2015). A total of 782 individuals across eleven year classes (2001–2011) were genotyped on this array, which was accessed through formal collaborations with CIGENE and Aquagen. After quality control (minor allele frequency (MAF) >0.02, SNP call rate >90%, Mendelian error control), a total of 98,948 assembly‐mapped polymorphic SNP and 750 individuals remained. A reduced set of informative markers was expected due to difference in continent of origin between the European salmon that were used to generate the array and the Tasmanian salmon of North American origin. Of the informative markers, 27,272 SNPs were found to be in perfect LD with another SNP in the set. These markers, on average, were within 53,809 bp of each other. This cleaned marker data, thinned for LD, was used to demonstrate the potential of within family selection through the utilization of genomic information into the breeding program (results not shown).

The characteristics of the markers were analyzed to determine what number were potentially needed for genomics selection. Through the assessment of a range of metrics including MAF distribution, LD statistics, and location, it was determined that a 50K array would be sufficient to create a useful primary genotyping tool. Initial discussions centered around the use of an array developed by another group breeding salmon from a geographically close Canadian region to the origin of Tasmanian salmon population. However, inspection of the selected markers revealed a much lower than optimal number of informative markers due to high numbers of monomorphic markers or markers in high LD. This led to the decision to develop a custom array.

### 3.2 Preliminary GWAS

Concurrently to exploring options for 50K genotyping tools, genome‐wide association studies (GWAS) were run using the cleaned 220K data (99K) to check for the presence of large effect QTL that could be used via marker assisted selection (MAS). None were found, indicating that MAS was not a viable approach for the selected traits within the breeding program. This complemented existing GWAS results completed previously using data from an Illumina iSelect SNP Chip (5568 markers) where a total of 1637 individuals from three year classes had been genotyped. After quality control filtering, a total of 2240 SNPs and 1574 individuals were used to undertake GWAS on the key traits with the breeding program (listed in Table 1). These traits were also analyzed using the 220K data, although there were limited phenotypes available for the set.

Markers from multiple significant regions were identified for each trait including a small number of regions that were found for multiple traits, which may indicate pleiotropic QTL (results not shown). These markers were consolidated to form a list of 270 potentially useful SNP for inclusion on any new array. This list included 25 markers from the Illumina platform that were not on the 220K array.

### 3.3 Custom 50K array design

The process to design and manufacture a custom 50K array was undertaken in 2015 through a partnership with Center for Aquaculture Technologies (CAT). The design process commenced with the aim to tag each segregating chromosome segment with at least one SNP to ensure even genome coverage. Consequently, the design process began by selecting all markers from the complete set (98K) not in high LD (*r* ^2^ < 0.2) with any other sets of markers to retain all possible distinct chromosome segments and the selected trait‐associated markers. All remaining markers then were placed in 100 kbp bins, scaffolded using the previously selected markers. The location of these previously selected markers, MAF and LD, was all then used to optimize the final set of markers. Markers with higher MAF (minimum MAF >0.02) and lower LD values were selected to optimize the final MAF distribution while ensuring that all chromosome segments were tagged with a marker.

The final set of markers contained 53,102 SNPs with a main set of 49,311 SNPs and supplementary set of 3791 SNPs. The markers in the main set had an average distance between markers of 45 kbp and a mean MAF of 0.19. The main list formed the preliminary framework for the final design. This allowed high priority markers to be duplicated and left space on the array for the 25 de novo markers from the Illumina platform, four sex markers and 96 pedigree markers (sourced from CAT). A comparison of the final list with the alternative 50K array found only 28,302 markers in common, confirming that that array would have delivered suboptimal data and results. The final in silico design of the custom Affymetrix array was carried out by CAT and resulted in 50,687 unique markers on a custom Affymetrix array.

### 3.4 Imputation analysis and low‐density assay development

The first set of 50K data, from 2889 individuals, was received in 2015. After quality control (MAF >0.02, SNP and individual call rates >90%), 45,840 SNPs were retained. Analysis of the LD in this dataset, supported by results from 220K data, revealed long extending linkage disequilibrium (LD). The trend showed moderate LD (*r* ^2^ = 0.2) extending to 500 kbp, indicating there was high potential for using of a low‐density assay as part of the genotype scheme. The use of low‐density assays as part of genomic selection strategies has been shown to contribute to a more cost‐effective approach (Houston et al., 2020). To assess the viability of a low‐density SNP panel, two sets of analyses were carried out. The first was an assessment of imputation accuracy by masking (setting to missing) sets of markers and then imputing these missing data using the marker data that remained. The imputed data were then compared to the original data and the accuracy of imputation assessed based on the number of correctly imputed genotypes. The second analysis looked at the impact of using the imputed data to calculate genomic breeding values (GEBV). The results from these analyses compared, via correlation, GEBV calculated using the original genotype data with GEBV calculated using imputed genotype data.

To complete both analyses, the 50K SNP dataset containing 45,840 SNPs and 2889 individuals from the 2012 and 2013 year classes was used. These data were combined with the corresponding set of markers in the 220K data (750 individuals). The imputation and GEBV accuracies were then assessed when predicting the 2013 year class (1703 individuals) using the remaining data (1936 individuals) from the 2001–2012 year classes. Two different sizes of low‐density assays were considered for implementation with SNP subsets of either 1000 (1K) or 3000 (3K) SNPs retained with all other markers masked. The choice of these densities reflected the genotyping options made available by CAT, using a targeted genotype‐by‐sequence (GBS) platform. An optimal set of markers for each density were selected to optimize genomic coverage, trait association, LD and MAF. Then, using the complete pedigree containing individuals from the 2001–2013 year classes and FImpute version 2.2 (Sargolzaei et al., 2014), the masked markers were imputed using family and population imputation. The proportion of correctly imputed genotypes was used to assess the accuracy of the imputation. High imputation accuracies were found with 88.4% and 95.8% of masked markers imputed correctly for the 1K and 3K SNP sets, respectively (Table 2).

**TABLE 2:** Imputation and “imputed” GEBV accuracies for genotyped individuals from the 2013 YC

No. of SNPs	Imputation accuracy	“Imputed” GEBV accuracy^a^	
		Harvest weight	Marine maturation
1000	0.8841	0.9778	0.9701
3000	0.9579	0.9960	0.9951

Note

Imputation accuracy is calculated as the proportion of correctly imputed genotypes.

^a^ Spearman's rank correlation coefficient was used to calculate the accuracy between GEBV from imputed genotypes (“imputed” GEBV) and GEBV using the original data.

The effect of imputation on GEBV accuracy was tested using two traits: harvest weight (no. of phenotypes = 13,157) and marine maturation (*n* = 33,754). Two models were fitted using ASReml. In the first, GEBV for harvest weight were estimated using a univariate model with cohort fitted as a fixed effect and, family and animal as random effects. In the second, GEBV for marine maturation were estimated using a multivariate model including maturation and weight measured in the marine and freshwater environment at 23 months (4 traits). This model included a mean trait effect and trait by cohort effects as fixed effects. The genetic effects were modeled as random by fitting trait by family (representing common environment plus nonadditive genetic effects) and trait by animal effects (representing additive genetic effects). Animal effects in both models were fitted using the H matrix, the relationship matrix based on both pedigree and genomic information (Aguilar et al., 2010), calculated in R (R Core Team, 2021) using standalone code. The resultant GEBV using imputed genotypes were correlated with GEBV calculated from the original called genotype data using BLUPF90 (Misztal et al., 2002). The accuracies obtained were extremely high and consistent for both traits (Table 2).

The accuracies obtained for both imputation and GEBV analyses confirmed that the use of a low‐density panel would be highly effective. As expected, using more markers on the lower density assay produced higher accuracies due to the improved genome‐wide coverage and increased tagging of the segregating chromosome segments. Given the known potential of GBS assays to produce higher amounts of missing data than SNP arrays and the lower cost associated with such assays, a custom 3K GBS assay was assessed as viable and the best approach.

The design of the initial GBS assay included 3073 markers selected to maximize imputation accuracy, ensure genome coverage and an appropriate MAF distribution. An additional 100 markers including sex and pedigree markers were added to this marker set. This marker list then went through in silico design. Any markers that failed were then iteratively replaced until sufficient genome coverage and successful imputation were achieved. At that point, the design was finalized with 3151 markers.

### 3.5 GEBV and EBV production

Standard breeding value production for the breeding program has always included four multitrait linear mixed models. These models remain the basis for the program and are now run using the Wombat software package (Meyer, 2007). All have the general form:

$$y=X\tau +Z_{n}u_{n}+Z_{g}u_{g}+e$$

where ***y*** is the vector of responses comprising multiple traits. The fixed Xτ and random $Z_{n}u_{n}$ effects reflect the nongenetic effects (such as cohort or maturation status), while ***e*** is a vector of residuals. It is assumed that $u_{n}∼N\left(0,\;G_{n}\right)$ and e∼N(0,R) and that they are uncorrelated where **G** *_n_* and **R** are the matrices of nongenetic and residual covariances across traits.

For the production of EBV utilizing only pedigree information, the genetic effects ***Z*** *_g_* ***u*** *_g_*, are assumed to be $u_{g}∼N\left(0,\;G_{p}⊗A\right)$, where **G** *_p_* is the matrix of genetic covariances across traits and **A** is the numerator relationship matrix. GEBV are produced when $u_{g}∼N\left(0,\;G_{p}⊗H\right)$, where **H** is the augmented genomic relationship matrix containing information from both the genomic relationship matrix (**G**) and the numerator relationship matrix (**A**) (Legarra et al., 2014). The **H** matrix was formed using the available genomic information for all individuals with quality checked genotypes available. For all GEBV runs after 2018, this was 45,997 markers both imputed and real. The genomic relationship matrix was scaled to ensure it could be inverted and a factor used to weight the proportion of polygenic (**A**) and genomic (**G**) information coming from the respective matrices (Christensen, 2012).

Acquired AGD resistance EBV or GEBV are calculated using the method from Schneeberger et al. (1992) using the breeding values for the AGD scores (EBV or GEBV) from the AGD model. All breeding values are scaled to be in units of genetic standard deviation. The index is then calculated using the selection pressures defined in Table 1.

### 3.6 Final scheme

**FIGURE 3:**
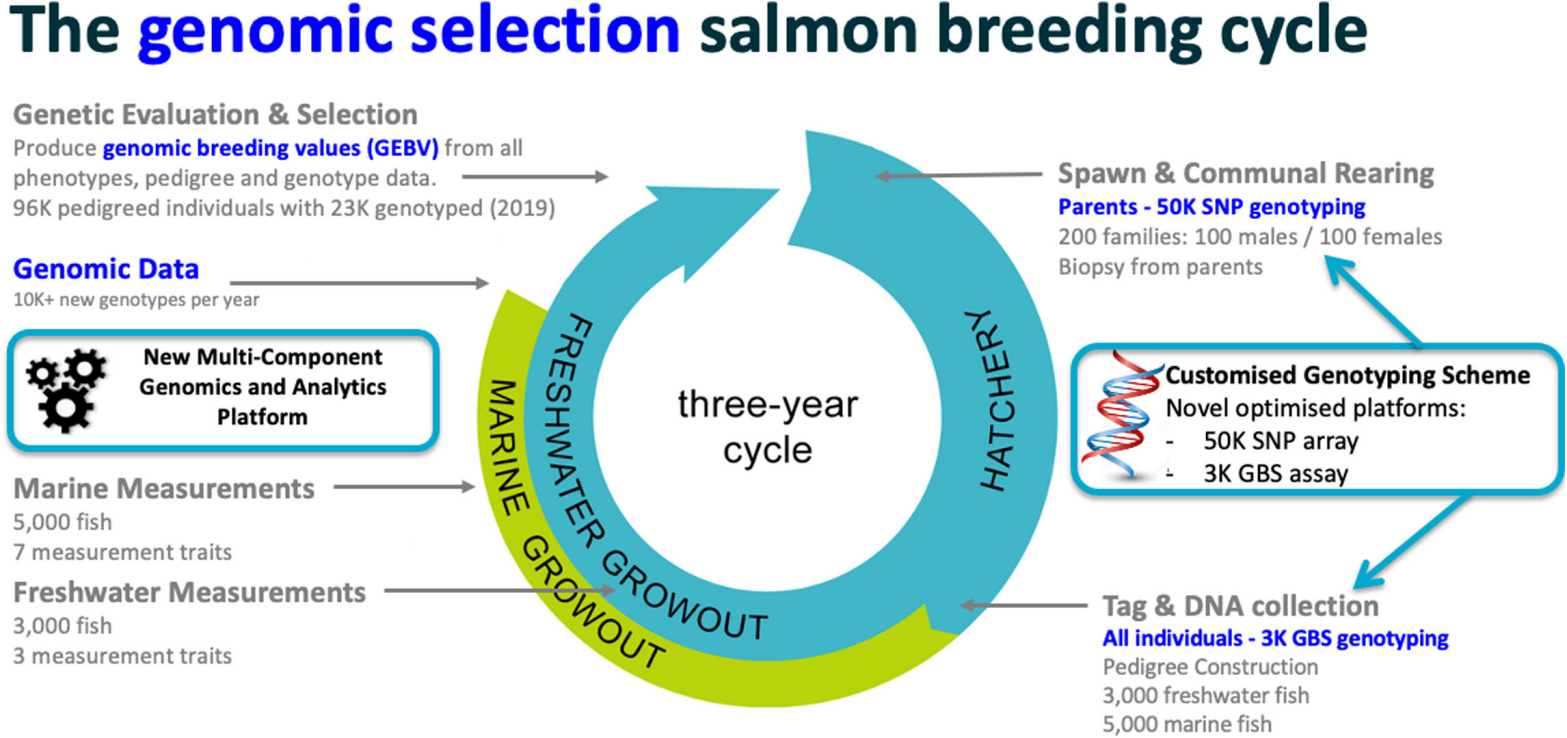
The genomic selection salmon breeding cycle. Blue text indicates the new components of the original breeding cycle and the cyan boxes contain the essential components developed to enable the commercial implementation of genomic selection. SNP, single nucleotide polymorphism; GBS, genotype‐by‐sequence

**FIGURE 4:**
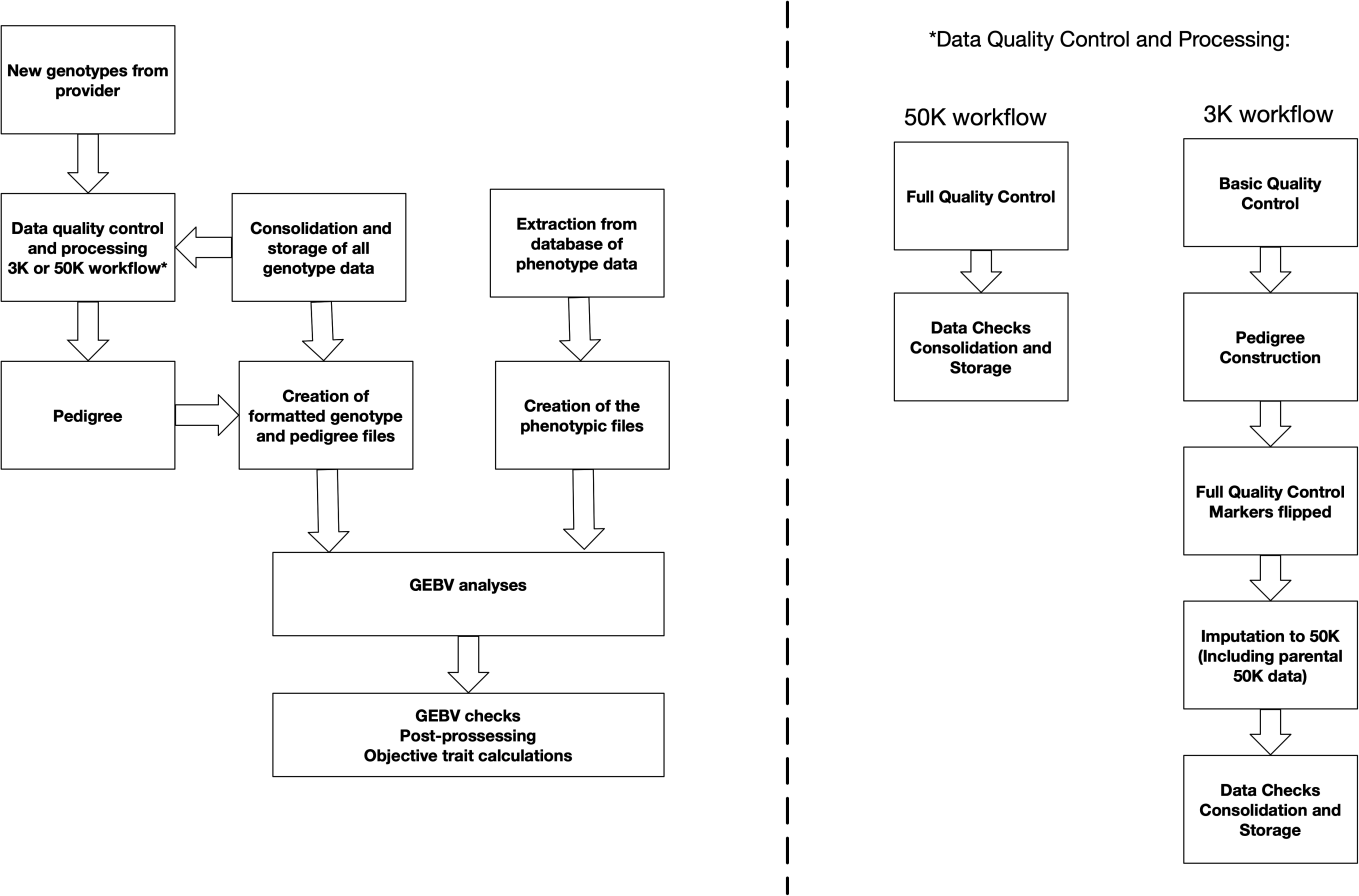
Genomic Selection Data Workflow. Basic Quality Control is the removal of any markers that failed quality control (MAF < 0.02 and typically less than 10% missing) but no individuals are removed to enable the maximum number of individuals to have parents assigned. Full Quality Control is the removal of both markers and individuals that failed quality control (MAF < 0.02 and typically less than 10% missing for both individuals and markers)

With the finalization of the cost‐effective genotyping scheme, full commercial implementation of genomic selection was achieved in 2018. The final genomic selection breeding cycle is shown in Figure 3. The key aspect of the implementation, evident when comparing Figures 1 and 3, is that very few operational aspects changed. The individuals previously biopsied for pedigree assignment are now genotyped on a low‐density array from which pedigree is established and genotype imputation occurs. The new component to the cycle is the inclusion of the genomics data. Underpinning this new aspect is the genomics and analytics platform that incorporates all the steps from processing of the genomic data (quality control, pedigree assignment, imputation and data integration) through to GEBV production through multivariate single‐step GBLUP. The data workflow is displayed in Figure 4.

Also supporting the efficient use of genomic data was the customized genotyping scheme. Designed specifically to be both cost‐effective and deliver optimal gains, the final scheme specified that all individuals in each new year class, totaling more than 8000 in the final scheme, are genotyped on the 3K GBS assay, while all parents, approximately 200 individuals, are genotyped on the 50K SNP array. A feature of the final scheme is that every marine tested and measured animal in every year class forms part of the training set, thereby providing strong links between genotypes and phenotypes and avoiding the need to define a training set as a subset of measured animals.

A key benefit of GBS technology is the ability to update the assay. The first version of the assay delivered useable data on 2372 markers. As a result, in consultation with CAT, a second version of the assay was developed with 820 new markers added to the informative markers from the first version. This is now delivering on average approximately 2500 consistently useable markers based on seven runs of the assay. The GBS data are imputed up to the standard set of 45,997 markers for consolidation with all other available SNP data for the purpose of GEBV production.

## 4 VALIDATION

To assess the success of the commercial implementation of genomic selection, the improvement in prediction/selection accuracy and the rate of improvement in genetic gain were assessed. The potential improvement in the rate of genetic gain was evaluated using the data available in 2019 by comparing the rates of gain using the GEBV to what would have been obtained using EBV. The increase in selection/prediction accuracy was examined by comparing the breeding values for broodstock at the time of spawning and after progeny information was available using the same models. These comparisons across multiple spawnings allows for a direct robust assessment of whether the use of genomic information improved the breeding program's ability to more accurately rank broodstock individuals as parents. This method provided a means to utilize the data from higher order relationships in the validation (such as aunt/uncle to niece nephew), and such relationships are numerous in this population due to the large family sizes. It differs from the classic approach of parent–progeny relationships, which uses first order relationships only.

### 4.1 Validation method and data

The length of the salmon breeding cycle means that validation information (e.g., progeny records) are not available until 2 years after spawning. The EBV and GEBV for the parents of three year classes were compared to the updated breeding values once progeny information was available two years later (e.g., breeding values for the 2016 year class parents after the 2016 and 2018 production runs). Three comparisons were made, one retrospectively using the parents of the 2015 year class individuals, and two more using parents of the 2016 and 2017 year class individuals. The updated breeding values (EBV or GEBV) from the run two years after spawning with the lowest standard errors were used as the closest estimate of the true breeding value. In most instances, this was the GEBV, but in all instances, the GEBV and EBV were more than 98% correlated. The ability of the index to accurately rank individuals was assessed in a similar manner to the individual breeding values. The index was calculated using the EBV or GEBV at the time of parent selection and then again using the updated breeding values. These were then compared to assess the accuracy of the index (GEBV or EBV based) to correctly rank the individuals for selection.

The phenotypic data used in the validation analyses are summarized in Table 3. More genotypic and phenotypic data were added each year. The 2019 GEBV run, which had the most data, included at least one phenotype from 96,171 individuals with 22,469 of these individuals genotyped. This included 31,573 (8574 genotyped) broodstock individuals and 60,661 (12,864 genotyped) marine‐environment tested individuals. Phenotypes from individuals (genotyped and nongenotyped) from all 554 families were included in the phenotypic dataset. The GEBV and EBV were calculated during the standard GEBV run process (Section 3.5) except for the 2015 results where the GEBV were calculated after the 2015 spawning was concluded. This occurred when the genotypic data became available postspawning with GEBV produced using only the phenotypic data that would have been available at prespawning to ensure equivalency to the processes that produced all the other GEBV data.

**TABLE 3:** Phenotype description and numbers availability

Phenotype		Description	Enviro.	2015 year class^a^		2016 year class^a^		2017 year class^a^		2018 year class^a^		2019 year class^a^	
				Total	Geno	Total	Geno	Total	Geno	Total	Geno	Total	Geno
AGD severity^b^	AGD1 AGD2 AGD3 AGD4 AGD5	Visual gill score (0–5)	Marine	36,135 23,442 31,912 8961 9569	1025 0 1954 0 0	40,865 23,442 36,209 10,679 9569	2865 0 2866 397 0	45,403 23,442 40,513 10,679 9569	3380 0 3381 397 0	50,240 23,442 43,669 10,679 9569	8204 0 5426 397 0	52,785 23,442 47,298 10,679 9569	10,653 0 9938 397 0
Flesh color		Astaxanthin content (mg/kg)	Marine	8418	512	9724	1088	11,271	1470	12,442	1762	13,441	2757
Flesh fat content		Percentage of fat in wet weight (%)	Marine	8170	508	9442	1074	10,948	1448	12,119	1740	12,519	2138
Harvest Weight		Head on gutted (HOG) weight (kg)	Marine	11,852	514	13,156	1089	14,721	1476	15,892	1768	16,885	2757
Maturation		Presence/absence of sexual maturity at 22 months	Marine	29,973	1809	33,754	2721	37,912	3236	41,268	6583	45,097	10,252
			Fresh	20,718	997	23,145	1919	25,572	2951	28,467	5836	31,315	8569
Weight at 23 months		Weight at 23 months	Marine	27,985	1807	31,751	2719	35,907	3234	39,261	6579	43,089	10,247
			Fresh	20,684	998	23,111	1920	25,538	2952	28,431	5835	31,279	8568
Weight at 30 months		Weight at 30 months	Marine	11,838	514	13,142	1089	14,707	1476	15,878	1768	16,871	2757
			Fresh	14,008	189	15,481	822	25,572	3234	17,201	1657	22,349	5697
Totals		No. of individuals		65,492	2978	72,660	4815	79,631	6362	87,367	14,075	96,171	22,469

Abbreviation: AGD, amoebic gill disease.

^a^ The total number of phenotypes (Total) or number of phenotypes with associated genotypes (Geno) available for the GEBV run in the specified year.

^b^ Intially, there were up to 5 AGD measurements due to repeated infections; since 2010, there is typically two representing the severity of AGD at first infection and at summer (peak) infection.

## 5 VALIDATION RESULTS

**FIGURE 5:**
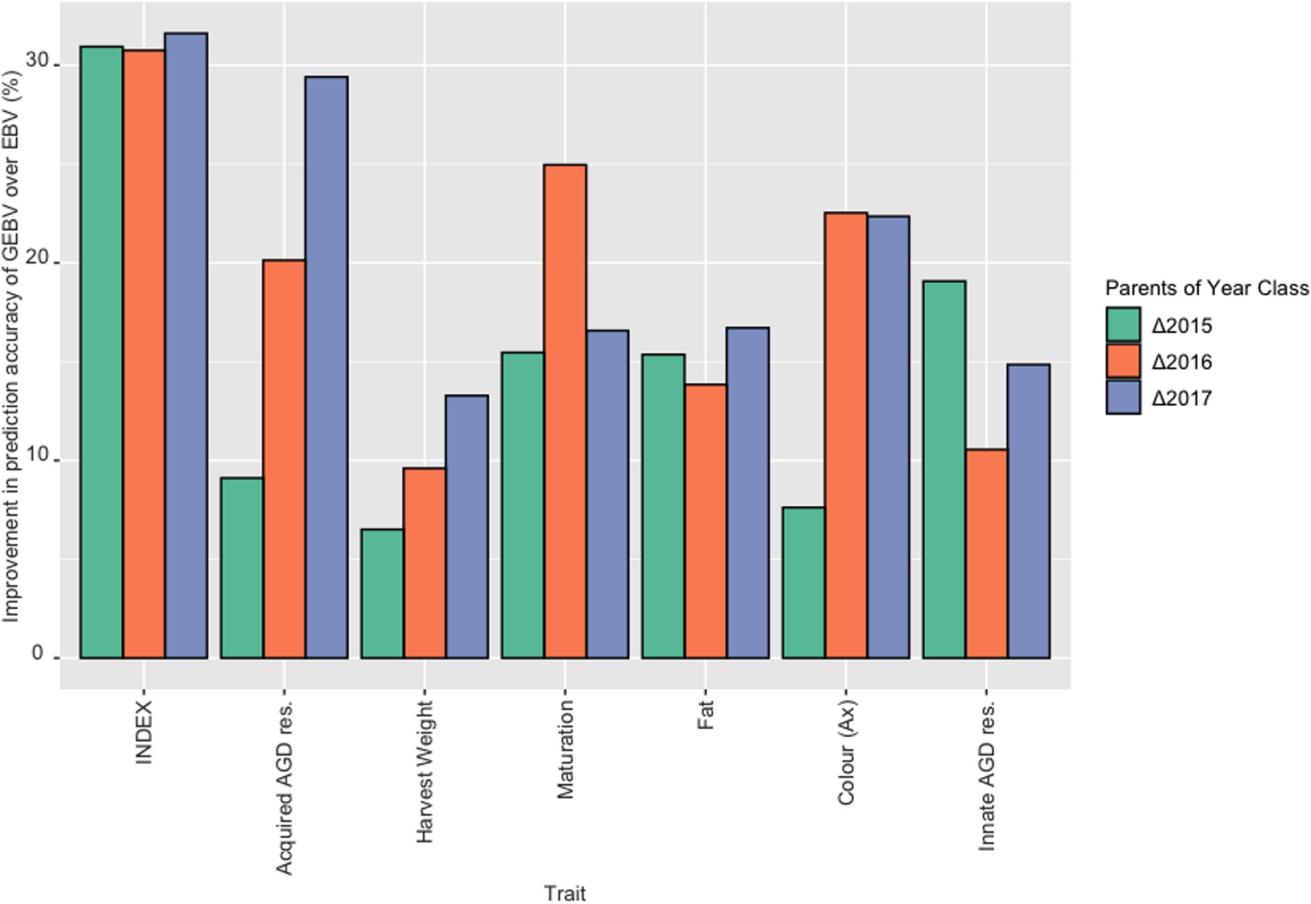
The percentage improvement in prediction accuracy for ranking broodstock individuals when using GEBV over EBV for the parents of three year classes

The results for the analyses examining the increase in selection/prediction accuracy are found in Figure 5 and Table 4. Increases in prediction accuracy were found for all traits, as well as the index, across the three sets of data compared. The highest increases in accuracy were for the index where the increase in accuracy was more than 30% when using GEBV. This included the retrospective 2015 year class analyses where EBV were used to select parents, indicating the result was not linked to the individuals selected or method used for selection. As both the phenotypic and genomic data increased, there has been an increase in prediction accuracy for the primary traits of harvest weight and acquired AGD resistance. The results for the secondary traits are more variable although still significant.

**TABLE 4:** Comparison of the prediction accuracies for the three years of spawning

Trait	2017–2019			2016–2018			2015–2017		
	*r*($\hat{T}$,G_17_)	r($\hat{T}$,E_17_)	Δ17	r($\hat{T}$,G_16_)	r($\hat{T}$,E_16_)	Δ16	Cor($\hat{T}$,G_15_)	Cor($\hat{T}$,E_15_)	Δ15
INDEX	0.6051	0.2891	0.3161	0.6249	0.3173	0.3075	0.6337	0.3242	0.3094
Acquired AGD res.	0.8198	0.5257	0.2941	0.8648	0.6635	0.2013	0.8337	0.7426	0.0911
Innate AGD res.	0.8769	0.7174	0.1485	0.7286	0.6231	0.1055	0.7355	0.5448	0.1907
Harvest weight	0.7905	0.6577	0.1328	0.8346	0.7386	0.0960	0.7564	0.6913	0.0651
Maturation	0.8351	0.6693	0.1657	0.8299	0.5803	0.2496	0.8591	0.7045	0.1546
Fat	0.9033	0.7362	0.1671	0.8074	0.6690	0.1384	0.7214	0.5678	0.1536
Color (Ax)	0.9156	0.6924	0.2235	0.8654	0.6401	0.2253	0.8656	0.7894	0.0762

Note

*r*($\hat{T}$,M_x_) is Spearman's correlation coefficient between M (G: GEBV or E:EBV) in year x with $\hat{T}$, the best estimate of the true breeding value represented by the GEBV/EBV with the highest accuracy in the comparison year (x+2) GEBV production run, for example, r($\hat{T}$,E_17_) is the correlation coefficient between the best estimate of the breeding values in 2019 compared to the EBV produced in 2017. The GEBV and EBV of progeny checked individuals are highly correlated (greater than 98%).

The rate of genetic gain improvement, based on the performance up to and including 2019, is shown in Table 4. Substantial improvements in the rate of genetic gain are observed for both primary traits, harvest weight and acquired AGD resistance, and the index, with increases of 19%, 54% and 109%, respectively. As expected, the magnitude of the gain improvement for secondary traits is low because low selection pressure is applied to these traits. However, there is still a 50% increase in genetic gain for Maturation and Flesh color through the use of GEBV compared to the EBV.

## 6 DISCUSSION

### 6.1 Genetic gains and value of genomic selection

The impetus for exploring the potential of genomic selection for the Tasmanian Salmon selective breeding program was a desire to increase the rates of genetic gain through enabling within family selection. Initially, a business case was built around increasing the rate of genetic gain for AGD resistance and economic analyses were done for that trait alone. That was assumed to be the main and perhaps only driver of value because other traits already benefited from moderate genetic correlations between marine and freshwater populations (Section 2). The increase in the rate of genetic gain has been highest for AGD resistance, as expected, with an increase in the annual rate of gain from 3.7% to 5.7%. This 54% increase is driven by the ability to make within family selections, resulting in an improved selection accuracy of up to 29% for this trait. This is a higher increase in prediction accuracy than previously reported for any AGD‐related trait (Aslam et al., 2020; Robledo et al., 2018). In commercial practice, the value of this gain is realized via fewer AGD treatments (freshwater bathes) during grow‐out and therefore a reduction in the growing cost. Conservatively, the value of that is estimated to be $AU1 million per year currently and is projected to accumulate to $AU5 million per year after 10 years.

The magnitude of the improvement in other traits and the extra value they provided was an unexpected benefit. Most notable is the improvement in total weight, where the annual rate of genetic gain increased from 3.6% to 4.3% per year (Table 5). This 19% increase in genetic gain reflects the 14% improvement in prediction accuracy when using GEBV compared to EBV for growth, a result consistent with the study results of Tsai et al. (2015). For the Tasmanian industry, the increase in genetic gain for growth is worth approximately AU$5 million per year at farm gate and exceeds the value of gains in AGD resistance.

**TABLE 5:** Rates of genetic gain for EBV and GEBV

Trait	EBV genetic gain (% per year)	GEBV genetic gain (% per year)
Acquired AGD res. (AGD treatment interval)	3.7	5.7
Harvest weight	3.6	4.3
Maturation	0.2	0.3
Color (Ax)	0.2	0.3
Fat	0.6	0.8
INDEX (in units of index)	1.31	2.75

The largest benefit from genomic selection is seen in the increase in the rate of gain in the index, which is the measure that combines all traits weighted by their assumed economic value (selection pressure for each trait shown in Table 1). There has been a twofold increase, which is a result that vastly exceed initial expectations. In practice, this is seen as a much higher level of discrimination between individuals of the same family when individual candidates are selected. It provides a means to better identify individuals with the desired mix of traits and, therefore, provides the breeder with far better options when multitrait selection is required.

### 6.2 Genotyping platforms

Decisions made about genotyping platforms, and the emphasis placed on obtaining optimal genotyping tools, have been instrumental to the successful implementation of genomic selection and are well recognized as essential to genomic selection (Abdelrahaman et al., 2017; Bangera et al., 2018; Houston et al., 2014; Yanez et al., 2016). The first of these was the decision to develop a Tasmanian population specific 50K genotyping array rather than use an existing product. There were expectations that an existing array should be used given the relatively small size of the Tasmanian breeding program when compared on a global scale. While the alternative product did have informative markers for the Tasmanian population, it was not ideal, with only 56% of markers uniquely informative, and the coverage of markers was sparse in places (Section 3.3). The design of the customized 50K array allowed optimal coverage, without large gaps, and it ensured all markers were informative that maximized the data available. This enabled more accurate breeding values and better gains by ensuring all chromosomal segments were captured.

The second important juncture was the decision to develop a low‐density array, to use that across all individuals in every year class, and to impute the 50K markers. Exploration of the use low‐density panels and imputation have been explored in multiple studies and shown to be useful across multiple aquaculture species (Tsai et al., 2017; Zenger et al., 2019). The adoption of such an approach allowed GEBV to be calculated for every measured individual and for all broodstock candidates. It avoided the need to subset a training population based on particular phenotypes and that has resulted in strong links between all phenotypes in all traits and genotypes. The breeding program has always used genotypes to determine pedigree (Section 2), and the cost of that has always been part of breeding program budgets. The opportunity was recognized to extend the scope of that genotyping to allow some level of genomic selection as part of the pedigree determination, and this direction was given to the genotyping provider to determine what was possible at an equivalent price to the pedigree assay. The genotyping platform decision was made in consultation with CAT, based on available technology at a specified price point. Ultimately, there was an ideal convergence of the characteristics of genotype platform needed for imputation (Section 3.4) and what was possible at the nominated price, using a GBS platform. This facilitated the consolidation of the final genotyping scheme (Section 3.5). The result was the ability to obtain genotypes at 40% of the cost of the 50K array. Collaborations with CAT, as the genotype provider, were essential to be able to utilize the current technology developments and to enable the logistical needs of the breeding program to be met.

### 6.3 Breeding program logistics

The implementation of genomic selection has been an incremental change to the breeding program operations rather than a complete re‐design. This is demonstrated by noting the similarity between the breeding cycle prior to genomic selection (Figure 1) and after genomic selection (Figure 3). There has been little change to the animal related activities. Candidate selection, family production, tagging, biopsies, and measurements all proceed exactly as they did before genomic selection. This is mostly due to the processes that were necessary for a DNA pedigree‐based breeding program being very similar to the processes needed for genomic selection.

The models used for genetic evaluation also had very little change. This was due to the use of the single‐step GBLUP methodology, which combines pedigree and genomic information. GEBV were calculated on the same scale as the former EBV, and therefore, index values and estimates of genetic progress were completely comparable both pre‐ and postgenomic selection. Consequently, the operational side of the breeding program saw little change to logistics and no change to the metrics used for selection decisions, only seeing the outcome which was far greater discrimination among selection candidates.

The significant changes to the breeding operations were related to the genotyping and the analyses of that data. This was one of the greater challenges to the implementation on the operational/industrial scale. New processes were added which were data‐intense and which required different skill sets, described in Section 3. These new activities needed to fit within the already tight timelines of breeding operations, necessitated by the seasonal aspects of the data flow and breeding cycle. That required careful planning to coordinate operations and efficient data pipelines. While the models and presentation of the breeding values did not have obvious differences, the computing requirements did. New software was needed, moving from ASReml to Wombat, and access to a high‐performance computer was necessary. The need for oversight on computing requirements is an ongoing issue given the continual increase in the numbers of genotypes and the need for increases in computing power that causes.

### 6.4 Commercial multipliers

There are clear opportunities to use genomic selection in commercial multipliers (Zenger et al., 2019). This opportunity was immediately apparent within the SALTAS program once genomic selection was implemented in the breeding population and commenced with the commercial production in 2019. Separate male and female multiplier lines are produced annually as terminal lines and the parents of these lines are individuals sourced from the breeding population. Therefore, sibling relationships exist between the multipliers and breeding population, which allows GEBV for multipliers to be calculated accurately and as part of the standard workflow of the breeding population GEBV calculations.

The focus, so far, has been on the male multipliers and the goal has been to maximize gains in harvest weight. All male multipliers are now tagged, biopsied, and genotyped on the 3K array, and GEBV are calculated for those individuals. There have been high additional gains due to the high intensity of selection possible due to the high fecundity of the males, with the top 1% of males being selected for commercial production. This provided an additional 11.4% gain for harvest weight in the male multipliers, which resulted in a 5.7% increase in the commercial population for the commercial production in 2019 (value is halved due to no further selection on the females). The increase in the commercial production could potentially have been 8.2% had all male candidates being sexually mature, a consequence of husbandry of broodstock. The value of the gain provided by applying genomic selection in the male multipliers has been very high. While not cumulative as it is in the breeding population, the value for the 2019 commercial production is estimated at 8 times more than the annual gain in the breeding population (AU$40 million).

## 7 CONCLUSION

Genomic selection has been implemented at a commercial scale in the Saltas selective breeding program. Operational implementation was first achieved in the breeding population with the production of the 2016 year class, and in 2018, the application was extended to the male multiplier population. Implementation was achieved through the development and use of a 50K population specific SNP array, together with a low‐density 3K array. Examination of imputation accuracies revealed the viability of a low‐density panel, and this has provided a cost‐effective genotyping scheme allowing all individuals in both the freshwater broodstock population and the marine sib‐test populations to be genotyped with the low‐density assay, with parents genotyped on the 50K SNP array.

Validation results show improvement in prediction accuracies for all traits at the point of broodstock selection, which results in clear and substantial increases in the rate of genetic gain for all traits. Increases are highest for AGD resistance. The increase in gains for harvest weight was half that for AGD resistance; however, the value of these gains is also substantial. Overall, the rate of gain for the index, combining all traits, was doubled through genomic selection.

## CONFLICT OF INTEREST

The authors declare no conflicts of interest.

## Data Availability

The data that support the findings of this study are available from SALTAS. Restrictions apply to the availability of these data, which were used under license for this study. Data are available from the authors with the permission of SALTAS.
